# U-shaped association between ultra-processed food intake and overactive bladder in US adults: a national cross-sectional study

**DOI:** 10.3389/fnut.2025.1618943

**Published:** 2025-08-06

**Authors:** Zufa Zhang, Li Chen, Long Lv, Wenkai Li, Bin Hou, Sheng Guan, Zuyi Chen, Danni He, Hongxuan Song, Weibing Sun, Feng Tian, Fengze Jiang, Sixiong Jiang

**Affiliations:** ^1^Affiliated Zhongshan Hospital of Dalian University, Dalian, China; ^2^Zhongshan Clinical College of Dalian University, Dalian, China; ^3^Key Laboratory of Microenvironment Regulation and Immunotherapy of Urinary Tumors of Liaoning Province, Dalian, China; ^4^Fuzhou Children's Hospital, Fujian Medical University, Fuzhou, China

**Keywords:** OAB, UPF, NHANES, U-shaped, association, RCS

## Abstract

**Background:**

The association between ultra-processed food (UPF) intake and overactive bladder (OAB) remains unclear. This study aimed to investigate the relationship between UPF consumption and the risk of OAB in U.S. adults.

**Methods:**

We conducted a cross-sectional analysis using data from 23,482 participants in the National Health and Nutrition Examination Survey (NHANES). UPF intake was assessed in g/day and kcal/day. Perform a natural logarithmic conversion of the UPF with *e* as the base. Weighted multivariable logistic regression was used to evaluate the association between UPF intake and OAB. Subgroup and interaction analyses were performed to assess effect modification. Restricted cubic spline (RCS) models were applied to explore potential non-linear relationships. Threshold effect analyses were conducted to identify inflection points.

**Results:**

UPF intake was positively associated with the risk of OAB in both continuous and categorical analyses. A U-shaped non-linear relationship between UPF intake and OAB risk was identified, with inflection points at 6.33 (g/day) and 5.73 (kcal/day). Subgroup analyses revealed stronger associations among women, smokers, and overweight or obese individuals. Interaction tests indicated significant modification effects by sex and smoking status.

**Conclusion:**

This study suggests a U-shaped association between specific levels of UPF intake and OAB risk among U.S. adults. Further prospective studies are needed to validate these findings and explore the underlying biological mechanisms.

## 1 Introduction

Overactive bladder (OAB) is a syndrome defined by symptoms such as urge incontinence, frequent urination, and nocturia, all of which significantly impair patients' quality of life and social functioning (1, 2). OAB emphasizes symptomatic diagnosis and focuses on the patient's subjective experience and quality of life impact (3). Epidemiological studies indicate that the prevalence of OAB is notably high among the global adult population, with its frequency rising progressively as age increases (4, 5). However, the etiology of OAB is not yet fully understood, and the potential influence of dietary factors on the development of OAB, in addition to the traditionally recognized risk factors, is gaining attention (6–8).

In the context of rapid changes in the global dietary structure, the proportion of intake of ultra-processed foods (UPFs) is rapidly increasing (9, 10). UPFs refer to foods that have undergone multiple industrial processes, are rich in additives, and are typically high in energy density and low in nutrient density (11). In the U.S. National Health and Nutrition Examination Survey (NHANES), researchers commonly categorize foods using the Nova classification system (12). The fourth group, ultra-processed foods, includes packaged snacks, sweetened beverages, ready-to-eat meals, and processed meat products (13). The proportion of UPFs consumed globally has continued to rise in recent years (14). Numerous studies have shown that UPF intake is strongly associated with an increased risk of obesity, fatty liver, cardiovascular disease, depression, and many other chronic diseases (15–19). However, studies on the relationship between UPF intake and OAB are still limited, and the underlying mechanisms and epidemiological characteristics are unclear.

Based on data from a nationally representative sample of U.S. adults, this study was designed to explore the relationship between ultra-processed food intake and OAB among U.S. adults. We hypothesized that the higher the intake of ultra-processed foods, the greater the risk of developing OAB. With this study, we hope to provide new epidemiological evidence for understanding the mechanism of the role of diet in the development of OAB. To provide a theoretical basis for the future development of OAB prevention and management strategies based on dietary interventions.

## 2 Methods

### 2.1 Study design and population

The present study followed the STROBE guidelines for cross-sectional studies. On one hand, the study protocol of NHANES received approval from the Ethical Review Board (ERB) of the National Center for Health Statistics (NCHS). Additionally, it was ensured that all study participants provided written informed consent before their participation. On the other hand, this study was a secondary analysis of the NHANES data and, therefore, did not undergo additional ethical review.

**Figure 1:**
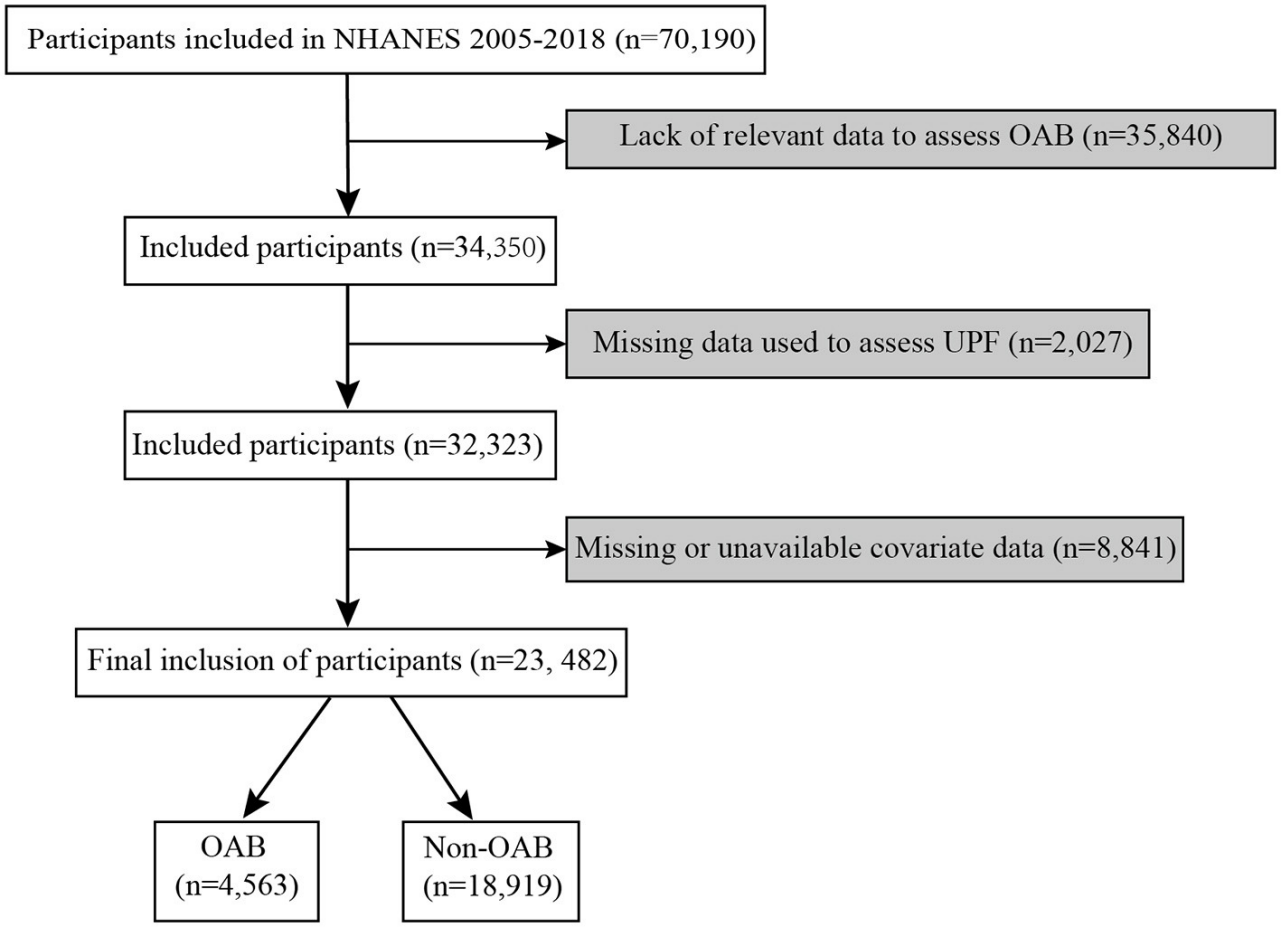
Flow chart of study population inclusion-exclusion.

The process of inclusion and exclusion in this study is shown in Figure 1. First, we identified 70,190 participants from 2005 to 2018 cycle. Participants who lacked data to assess OAB were subsequently excluded (*n* = 35,840). Participants who were unable to assess UPF were further excluded (*n* = 2,027). In addition, missing covariates were excluded (*n* = 8,841) after covariates were identified by a priori examination and clinically relevant criteria. Ultimately, a total of 23,482 participants were included in this study.

### 2.2 Assessment of OAB

OAB was the exposure variable in this study. Specifically, we quantitatively scored the OAB using standardized questionnaires in NHANES (KIQ044, KIQ450, and KIQ480) (20). Detailed scoring criteria can be found in Supplementary Table S1. Participants with a total score of 3 or more were defined as patients with overactive bladder, a feasible and validated protocol based on available data from the International Continence Society and NHANES (21).

### 2.3 Assessment and handling of ultra-processed foods

Based on prior experience, each food item reported by participants in NHANES was classified according to the degree of industrial processing using the Nova classification system (22). After recording, all food items were assigned to four Nova groups (23), with detailed descriptions of each group shown in Supplementary Table S2. In this study, the exposure of interest was the daily energy intake of UPF, which belonged to group IV.

Since the UPF is skewed, we performed a logarithmic natural transformation with *e* as the base. The ln-transformed UPF was obtained and used as a continuous variable for subsequent analyses. In addition, we grouped the UPFs according to their quartiles, with the lowest quartile serving as the reference group to explore the impact of high levels of UPFs. In addition, we calculated and reported both kcal and gram to enhance the comparability of results.

### 2.4 Covariates

The selection of covariates was based on a priori examination and clinical considerations (24). The main ones included sociodemographic variables, comorbidities, laboratory results, and other conditions such as diet. Age, gender, race and ethnicity, marriage, education, poverty income ratio (PIR), and body mass index (BMI) were the sociodemographic covariates we routinely considered. Comorbidities were considered for diabetes mellitus and hypertension. Based on clinical considerations, we chose serum creatinine (SCR) and serum uric acid (SUA) as confounding laboratory indicators. Due to dietary complexity, we chose the Healthy Eating Index (HEI) to assess the overall diet of the participants. In addition, we considered the effects of smoking and alcohol consumption, which are known risk factors for OAB (7, 25).

### 2.5 Statistical analysis

According to NHANES guidelines, corresponding weights were considered in this study. Categorical variables are expressed as frequencies (%). Continuous variables are expressed as mean ± standard deviation (SD). When continuous variables are skewed, we use the median (IQR) representation. The *t*-test for continuous variables and a chi-square test for categorical variables were used to analyze the differences between the OAB and non-OAB groups. Using weighted multivariate logistic regression, three models were constructed to assess the association between UPF and OAB. Subgroup analyses and interaction tests were used to assess the effects of different subgroups and inter-subgroup interactions. Restricted cubic spline curve (RCS) was used to assess the non-linear relationship between UPF and OAB. Threshold effects analyses further explored the inflection point values under total population and significant stratification. In the multivariate logistic regression models, we used untransformed UPF variables (g/day and kcal/day) to facilitate clinical interpretation of the odds ratios. In contrast, due to the significant positive skewness of the distribution of UPF intake (skewness = 2.51 and 1.94), the specific pre- and post-transformation results are shown in Supplementary Table S3. Natural logarithmic transformations were therefore used in the RCS and the threshold effects analyses in order to normalize the distributions and capture potential non-linear trends. All statistical analyses were performed using DecisionLnnc 1.0 software (26), R software (version 4.4.1), and Empower software (www.empowerstatas.com). A two-sided *P*-value of < 0.05 was regarded as statistically significant.

## 3 Results

### 3.1 Baseline characteristics

We performed baseline characterization according to whether or not we had OAB (Table 1). There were 23,482 participants enrolled in the study. Their mean age was 47.35 years. Median UPF was 889.20 (g/day) or 714.00 (kcal/day). Participants with OAB were more likely to be older, female, more educated, hypertensive, diabetic, and have a smoking habit. In addition, participants with OAB were likely to have a higher SCR, SUA, and BMI. Interestingly, among participants with OAB, we observed a higher proportion of non-drinkers, which may be related to voluntary alcohol avoidance to alleviate urinary symptoms.

**Table 1:** Baseline characteristics of the study population.

**Characteristic**	**Overall (*N* = 23,482)**	**Non-OAB (*N* = 18,919)**	**OAB (*N* = 4,563)**	***P*-value**
Age, years	47.35 ± 16.68	45.40 ± 16.09	58.20 ± 15.73	< 0.001
**Sex**, ***n*** **(*****P*****%)**				
Male	11,870 (49.81)	9,961 (51.84)	1,909 (38.48)	< 0.001
Female	11,612 (50.19)	8,958 (48.16)	2,654 (61.52)	
**Race**, ***n*** **(*****P*****%)**				
Mexican American	3,664 (7.88)	2,991 (8.03)	673 (7.06)	< 0.001
Other Hispanic	2,095 (4.74)	1,672 (4.73)	423 (4.78)	
Non-Hispanic White	11,014 (71.22)	9,006 (71.78)	2,008 (68.08)	
Non-Hispanic Black	4,712 (10.01)	3,477 (8.99)	1,235 (15.69)	
Other Race	1,997 (6.15)	1,773 (6.47)	224 (4.39)	
**Education level**, ***n*** **(*****P*****%)**				
Above high school	12,545 (62.08)	10,641 (64.30)	1,904 (49.68)	< 0.001
High school or GED	5,398 (22.49)	4,313 (22.03)	1,085 (25.07)	
Less than high school	5,539 (15.43)	3,965 (13.67)	1,574 (25.25)	
**Marital status**, ***n*** **(*****P*****%)**				
Married	12,286 (56.59)	10,096 (57.21)	2,190 (53.11)	< 0.001
Never married	4,169 (17.32)	3,619 (18.44)	550 (11.11)	
Living with partner	1,886 (8.01)	1,622 (8.31)	264 (6.33)	
Other	5,141 (18.08)	3,582 (16.05)	1,559 (29.45)	
**Alcohol**, ***n*** **(*****P*****%)**				
Yes	20,375 (89.57)	16,571 (90.22)	3,804 (85.91)	< 0.001
No	3,107 (10.43)	2,348 (9.78)	759(14.09)	
**Hypertension**, ***n*** **(*****P*****%)**				
Yes	10,146 (38.34)	7,207 (34.59)	2,939 (59.26)	< 0.001
No	13,336 (61.66)	11,712 (65.41)	1,624 (40.74)	
**Diabetes**, ***n*** **(*****P*****%)**				
Yes	4,530 (14.70)	2,960 (12.14)	1,570 (29.01)	< 0.001
No	18,952 (85.30)	15,959 (87.86)	2,993 (70.99)	
**Smoke**, ***n*** **(*****P*****%)**				
Never	12,629 (53.97)	10,435 (55.13)	2,194 (47.55)	< 0.001
Former	5,899 (25.41)	4,508 (24.47)	1,391 (30.68)	
Current	4,954 (20.61)	3,976 (20.40)	978 (21.77)	
HEI-2015	50.75 ± 13.67	50.75 ± 13.65	50.77 ± 13.79	0.739
SCR, mg/dl	0.90 ± 0.33	0.89 ± 0.32	0.92 ± 0.38	0.778
SUA, mg/dl	5.45 ± 1.39	5.45 ± 1.38	5.47 ± 1.45	0.993
BMI	28.94 ± 6.75	28.56 ± 6.50	31.04 ± 7.64	< 0.001
PIR	3.06 ± 1.63	3.15 ± 1.63	2.58 ± 1.59	< 0.001
UPF intake, g/day	889.20 (483.00–1,480.53)	903.62 (493.43–1,503.00)	800.50 (428.73–1,358.40)	< 0.001
UPF intake, kcal/day	714.00 (381.00–1,193.00)	732.00 (392.00–1,217.00)	621.00 (317.00–1,057.00)	< 0.001

OAB, overactive bladder syndrome; UPF, ultra-processed food; HEI, healthy eating inde; SCR, serum creatinine; SUA, serum uric acid; BMI, body mass index; PIR, poverty income ratio.

### 3.2 Daily intake of UPF was positively correlated with OAB

Three weighted multivariate logistic regression models were used to examine the association between UPF and OAB (Table 2). The fully adjusted model was the focus of our attention. For both observed continuous and categorical variables, we reported two units of UPF. For continuous variables, UPF and OAB were positively associated when measured in g/day (OR = 1.10, 95% CI: 1.02–1.15). UPF was also positively associated with OAB when measured in kcal/day (OR = 1.05, 95% CI: 1.01–1.10). For categorical variables, the fourth quartile of UPF intake (g/day) was positively associated with the risk of OAB compared to the first quartile (OR = 1.35, 95% CI: 1.18–1.54). When expressed in kcal/day, UPF's highest quartile Q4 remained positively associated with OAB (OR = 1.24, 95% CI: 1.08–1.42).

**Table 2:** Weighted multivariate logistic regression modeling of ultra-processed food intake and overactive bladder syndrome.

**Variable**	**Adjusted OR (95% CI)**, ***P*****-value**		
	**Model 1**	**Model 2**	**Model 3**
UPF intake, g/day	1.11 (1.06–1.67), < 0.001	1.10 (1.05–1.15), < 0.001	1.10 (1.05–1.15), 0.0002
**UPF (g) quartiles**			
Q1	Ref.	Ref.	Ref.
Q2	0.99 (0.87–1.12), 0.8806	0.98 (0.86–1.12), 0.8094	0.99 (0.86–1.13), 0.8405
Q3	1.20 (1.05–1.36), 0.0074	1.18 (1.03–1.35), 0.0146	1.18 (1.03–1.35), 0.0182
Q4	1.41 (1.23–1.61), < 0.001	1.36 (1.20–1.55), < 0.001	1.35 (1.18–1.54), < 0.001
UPF intake, kcal/day	1.05 (1.00–1.09), 0.0418	1.05 (1.00–1.09), 0.0377	1.05 (1.01–1.10), 0.0252
**UPF (kcal) quartiles**			
Q1	Ref.	Ref.	Ref.
Q2	1.04 (0.93–1.17), 0.5009	1.07 (0.95–1.20), 0.2823	1.07 (0.95–1.21), 0.2402
Q3	1.10 (0.96–1.27), 0.1681	1.13 (0.97–1.32), 0.1021	1.14 (0.98–1.33), 0.0854
Q4	1.26 (1.10–1.44), 0.0011	1.23 (1.08–1.40), 0.0026	1.24 (1.08–1.42), 0.0021

Adjusted for age, sex, race and ethnicity, BMI, PIR, education level, marital status, smoking, alcohol, diabetes, hypertension, HEI-2015, SCR, SUA. Odds ratios (ORs) in this table are derived from logistic regression models using untransformed UPF intake, expressed in g/day and kcal/day, respectively.

UPF, ultra-processed food; HEI, healthy eating index; SCR, serum creatinine; SUA, serum uric acid; BMI, body mass index; PIR, poverty income ratio.

### 3.3 Subgroup analysis and interaction tests

**Figure 2:**
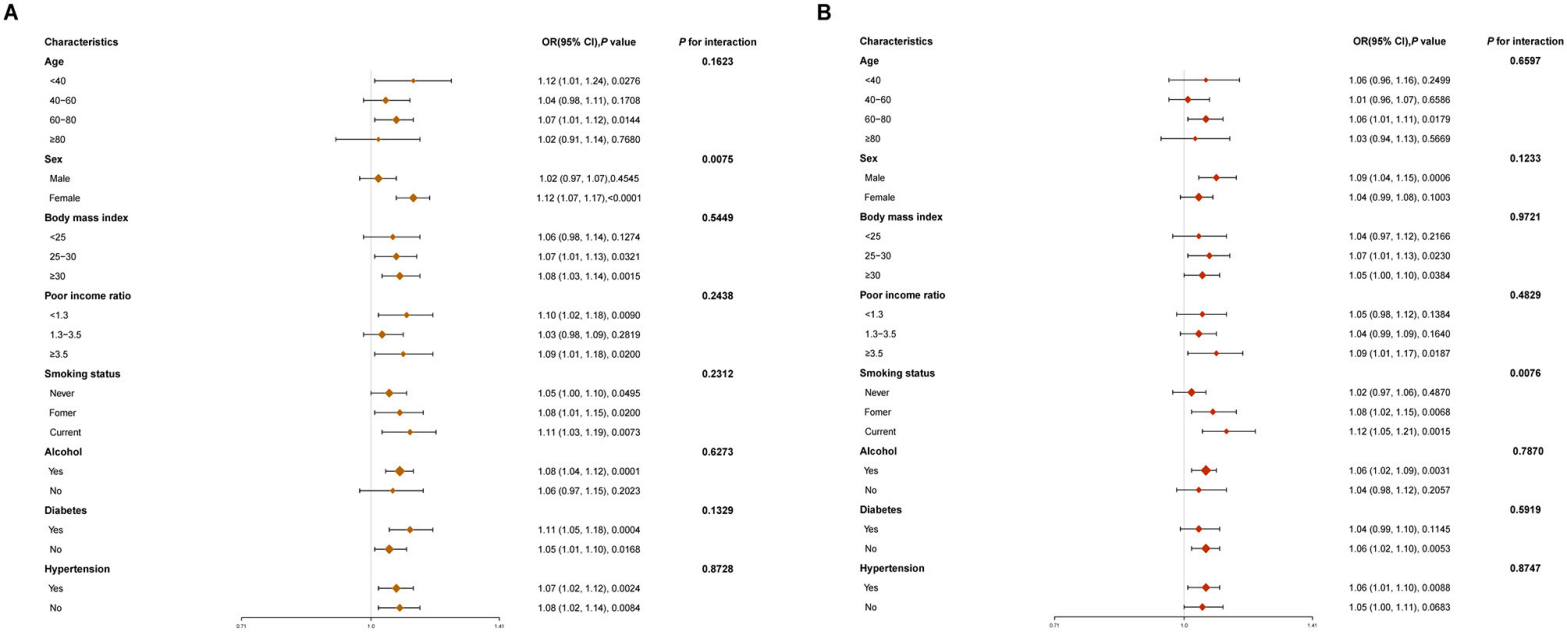
Forest plot of subgroup analyses of UPF and OAB associations. **(A)** UPF in g/day; **(B)** UPF in kcal/day. The original UPF intake is used here to facilitate the clinical interpretability of the odds ratios (OR).

Regarding the association between UPF and OAB, we conducted further subgroup analyses and interaction tests based on fully adjusted models (Figure 2). When analyzed in terms of g/day (Figure 2A), we found stronger associations for females, age < 40 or 60–80 years, obesity or overweight, and smokers and drinkers (all *P* < 0.05). In addition, sex was a significant modifier of UPF and OAB risk (*P* for interaction = 0.0075). When analyzed in terms of kcal/day, significant differences in the positive associations for UPF and OAB were similarly found between some subgroups (Figure 2B). However, we found smoking to be a significant modifier of UPF and OAB risk at this point (*P* for interaction = 0.0076).

### 3.4 Specific levels of UPF intake showed a unique U-shaped non-linear association with OAB

**Figure 3:**
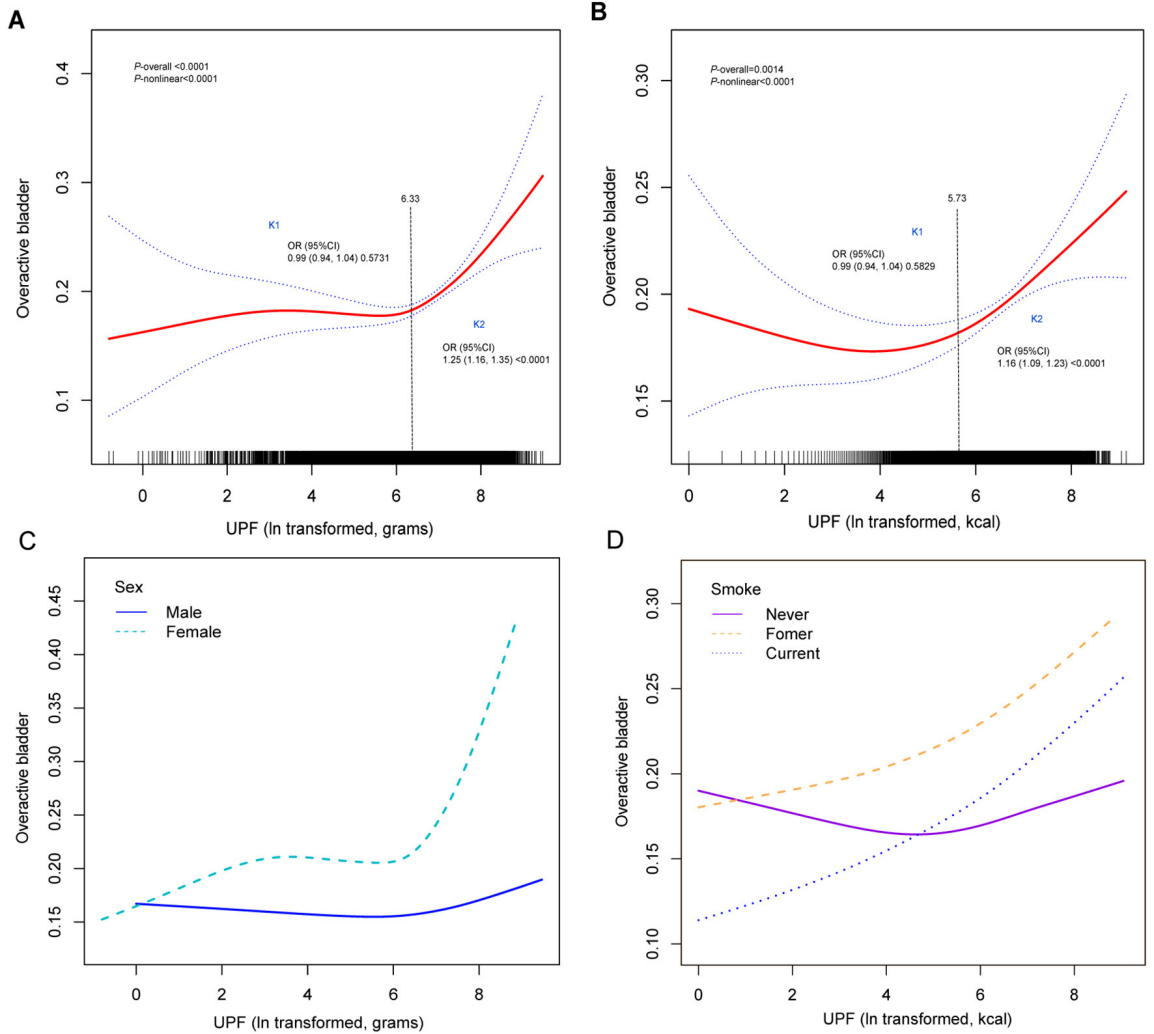
Overall and stratified RCS curve analyses of UPF (ln transformed) and OAB associations. **(A)** Overall RCS, ln UPF in g/day; **(B)** overall RCS, ln UPF in kcal/day; **(C)** sex-stratified RCS, ln UPF in g/day; **(D)** smoking-stratified RCS, ln UPF in kcal/day. For restricted cubic spline, ln-transformed UPF intake was used.

To explore whether there was a non-linear association between the relationship between UPF and OAB, we used RCS analysis (Figure 3). When analyzed by g/day (Figure 3A), we found a U-shaped non-linear association between UPF intake and OAB (*P*-overall < 0.0001; *P* for non-linear < 0.0001). When analyzed by kcal/day (Figure 3B), we found the same U-shaped non-linear association between UPF intake and OAB (*P*-overall = 0.0014; *P* for non-linear < 0.0001). Based on the results of the interaction test, to examine the significant effect of modification effects on the association between UPF and OAB. We further conducted stratified RCS analyses based on the aforementioned potential modifier effect variables. We found that for g/day UPF, the risk of having OAB behaved differently for men and women, and it appeared that there was a significant inflection point and higher risk for women (Figure 3C). Whereas for kcal/day UPF, it seemed that smoking presented a higher risk for both UPF and OAB risk, both for previous and current smoking (Figure 3D).

### 3.5 Analysis of overall threshold effects and stratified threshold effects for UPF and OAB associations

To explore potential inflection points in the UPF and OAB associations, we further used threshold effects analyses (Table 3). Overall, both UPF and OAB showed non-linear associations. Therefore, it was necessary to further analyze their inflection point values. The inflection point value was 6.33 for the U-shaped association of UPF and OAB in g/day (Figure 3A) and 5.73 when expressed in kcal/day (Figure 3B). The inflection points identified in the threshold effects analysis were 6.33 g/day and 5.73 kcal/day, corresponding to actual UPF intakes of ~560 g/day and 307 kcal/day, respectively. For the association of UPF and OAB in g/day, males (*P*-overall = 0.0195; *P* for non-linear = 0.002).) and females (*P*-overall = 0.0003; *P* for non-linear < 0.001) both showed non-linear associations, with specific inflection point values shown in Table 3. For the association between UPF and OAB for kcal/day, the association between previous smoking (*P*-overall = 0.0075; *P* for non-linear = 0.149) and current smoking (*P*-overall = 0.0125; *P* for non-linear = 0.168) both showed linear associations.

**Table 3:** Analysis of total and stratified threshold effects of the association between ultra-processed foods and overactive bladder.

**Exposure**	**Classification**	**<*K* slope 1**,	**≥*K* slope 2**,	**Inflection point (*K*)**	***P* for overall**	***P* for non-linear**
		**OR(95%CI)**, ***P*****-value**				
UPF (g)	Total	0.99 (0.94, 1.04) 0.5731	1.25 (1.16, 1.35) < 0.0001	6.33	< 0.0001	< 0.0001
	Male	0.95 (0.87, 1.04) 0.2683	0.99 (0.93, 1.05) 0.8003	6.16	0.0195	0.002
	Female	0.22 (1.10, 1.34) < 0.0001	1.44 (1.27, 1.63) < 0.0001	6.49	0.0003	< 0.001
UPF (kcal)	Total	0.99 (0.94, 1.04) 0.5829	1.16 (1.09, 1.23) < 0.0001	5.73	0.0014	< 0.001
	Never	0.90 (0.82, 1.00) 0.0502	1.09 (1.02, 1.17) 0.0109	4.48	0.3597	0.010
	Former	1.07 (1.00, 1.14) 0.0363	1.61 (0.95, 2.73) 0.0779	7.26	0.0075	0.149
	Current	1.05 (0.95, 1.16) 0.2987	1.27 (1.03, 1.57) 0.0283	6.67	0.0125	0.168

For the threshold effects analysis, which facilitates the observation of non-linear associations, the ln-transformed UPF was used. The corresponding raw UPF intake values at the inflection point were ~560 g/day and 307 kcal/day, respectively.

## 4 Discussion

In this nationally representative cross-sectional study, we report on two units of UPF based on UPF. We found that higher UPF intake was associated with a higher prevalence of overactive bladder OAB, and there was a significant positive association between them. Subgroup analyses showed that this positive association was particularly pronounced in females, specific age groups, overweight or obese individuals, smokers, and alcohol drinkers, suggesting that these factors may modify the relationship between UPF and OAB. In addition, there appeared to be potential modifying effects of gender and smoking, and RCS analyses identified a unique U-shaped non-linear association between specific levels of UPF intake and OAB risk. Stratified RCS analyses further highlighted that the UPF-OAB association was more pronounced in women and the smoking population. Finally, threshold effects analyses identified specific inflection points for g/day and kcal/day measurements beyond which the risk of OAB increased significantly, thus supporting the U-shaped trend observed in the overall analysis. It was also revealed that UPF and OAB showed a non-linear association across gender, whereas UPF and OAB in the smoking population showed a linear association, thus supporting the different trends observed in the stratified analyses.

Interestingly, the median UPF intake in the OAB group appears lower than that in the non-OAB group (800.5 vs. 903.6 g/day). This seemingly contradicts the positive association observed in regression models. However, baseline comparisons in Table 1 are unadjusted and reflect raw group differences without accounting for potential confounders such as age, sex, comorbidities, and lifestyle factors. In contrast, the multivariable regression analysis adjusts for these variables and reveals the independent effect of UPF on OAB risk. Furthermore, the presence of a U-shaped non-linear association—confirmed through restricted cubic spline analysis—indicates that both low and high UPF intake may carry elevated risks, with the lowest risk occurring at moderate intake levels.

Previous research has indicated that a higher intake of UPF is linked to an elevated risk of metabolic syndrome, type 2 diabetes, and obesity, all of which are established risk factors for bladder dysfunction (27–29). This is consistent with our finding that higher UPF intake is positively associated with an increased risk of OAB in terms of trend. Interestingly, our study uncovered a novel U-shaped non-linear relationship between UPF intake and the risk of OAB, a pattern that has seldom been reported in the existing literature. Most existing studies posit a linear relationship between UPF consumption and disease, highlighting that greater intake of ultra-processed foods is linked to a deterioration in health status (30, 31). However, our findings suggest that both very low and excessive UPF intake may be harmful, whereas moderate UPF intake is associated with the lowest risk of OAB. This unexpected U-shaped pattern may reflect the complex interplay between nutritional adequacy and the harmful effects of food processing. In addition, subgroup analyses in our study showed that the positive association between UPF intake and OAB was more pronounced in women, smokers, overweight/obese individuals, and specific age groups. Hormonal fluctuations, particularly the deficiency of estrogen in postmenopausal women, can compromise the integrity of the bladder mucosa, thereby worsening the symptoms of OAB (32). Smoking has been shown to impair bladder epithelial defense mechanisms and promote chronic low-grade inflammation, potentially amplifying the adverse effects of taking UPF (33). In addition, overweight and obesity increase intra-abdominal pressure and alter bladder function, while age-related changes in bladder innervation and muscle contractility may further alter susceptibility to dietary exposure (34, 35). Together, these observations emphasize the importance of considering individual susceptibility when assessing dietary risk factors for OAB.

The observed U-shaped relationship between UPF intake and OAB risk may have several plausible biological mechanisms. Excessive consumption of UPF, typically high in salt, saturated fats, artificial additives, and preservatives, has been found to induce chronic systemic inflammation and oxidative stress (36–38). These processes undermine the integrity of the bladder-urinary epithelium and facilitate detrusor overactivity, ultimately contributing to the onset of OAB symptoms (39). On the other hand, low UPF intake may reflect inadequate total energy intake and nutritional deficiencies, which are critical for tissue repair and the maintenance of functional integrity of the bladder wall (40, 41). Thus, poor nutritional status associated with very low UPF intake may also contribute to OAB. Taken together, these mechanisms highlight the complex interplay of diet, systemic health, and individual biological susceptibility in the pathogenesis of OAB.

This study has several strengths: first, we used a large nationally representative sample. Second, weighted analyses were used to adjust for multiple confounders. Third, non-linear relationships and inflection points were explored using RCS and threshold effects analysis. However, some limitations must also be acknowledged: first, the cross-sectional design does not allow for the inference of causality. Second, some of the information was self-reported and there may be some information bias. Third, there may be other potential confounders that were not fully adjusted for.

## 5 Conclusions

In conclusion, our study suggests a unique ‘U' shaped relationship between specific levels of UPF and risk of bladder OAB in US adults. These findings highlight the complex role of dietary patterns in bladder health and underscore the need for balanced and moderate food processing exposure in dietary recommendations. Further prospective studies are needed to validate these findings and explore the underlying biological mechanisms.

## Data Availability

Publicly available datasets were analyzed in this study. This data can be found here: https://www.cdc.gov/nchs/nhanes/.
