# Supplementary material for: U-shaped association between ultra-processed food intake and overactive bladder in US adults: a national cross-sectional study
Source: Front Nutr. 2025 Aug 6;12:1618943. doi: 10.3389/fnut.2025.1618943 (PMC12364681; doi:10.3389/fnut.2025.1618943)
Supplement: Supplementary file 1 [file Table_1.docx]

**Table S 1.** Criteria for conversion of symptom frequencies recorded in NHANES and OABSS scores

| **According to NHANES score** | **According to OABSS score** |
| --- | --- |
| Urge urinary incontinence frequency | Urge urinary incontinence score |
| Never | 0 |
| Less than once a month | 1 |
| A few times a month | 1 |
| A few times a week | 2 |
| Every day and/or night | 3 |
| Nocturia frequency Nocturia score | Nocturia frequency Nocturia score |
| 0 | 0 |
| 1 | 1 |
| 2 | 2 |
| 3 | 3 |
| 4 | 3 |
| 5 or more | 4 |

**N**ote: Individuals with a total score of ≥3 points are considered to be diagnosed with overactive bladder.

**Table S 2.** NOVA group classification table adapted from Monteiro et al. 2019[1].

| **Group and Definition** | **Examples** |
| --- | --- |
| 1. Unprocessed or minimally processed Foods  Natural foods that are the edible parts of plants or animals. Only alterations are to processes designed to make them suitable for storage, safer or easier to consume, such as drying, crushing, or freezing. | Natural vegetables, dried or fresh fruits, nuts, fresh frozen or dried meat and fish, fresh and dried eggs, yoghurt without sugar |
| 2. Processed Culinary Ingredients  Substances derived from group 1 foods from processes such as milling, pressing, and refining. These foods may be depleted in some nutrients and more energy dense. These foods are generally used as ingredients. | Plant oils, butter, lard, maple syrup, honey, salt |
| 3. Processed Foods  These are made by adding salt, oil, sugar, or other substances to group 1 or 2 foods. Processes include cooking and preserving methods. These foods generally have 2 or 3 ingredients and are recognizable as modified group 1 foods. | Canned or bottled vegetables or legumes, processed meats such as ham or smoked fish, freshly baked bread, simple cheeses |
| 4. Ultra-processed Foods  Formulations of ingredients, generally produced by industrial techniques and processes such as fractioning, hydrolysis and hydrogenation. Resulting products are generally energy dense and highly palatable. | Carbonated soft drinks, candy, package breads, cookies and biscuits, sweetened breakfast cereals, processed meats such as burgers and hot dogs, packaged soups, noodles, and desserts. |

1. Monteiro CA, Levy RB, Claro RM, Castro IR, Cannon G: **A new classification of foods based on the extent and purpose of their processing**. *Cadernos de saude publica* 2010, **26**(11):2039-2049.

**Table S3.** Skewness and kurtosis before and after UPF conversion.

|  | Original | Ln transformed |
| --- | --- | --- |
| **UPF（gram/day）** |  |  |
| Skewness | 2.51 | -1.39 |
| Kurtosis | 11.56 | 3.98 |
| **UPF（kcal/day）** |  |  |
| Skewness | 1.94 | -1.68 |
| Kurtosis | 7.11 | 4.51 |
